# Large osteochondral defect in the lateral femoral condyle reconstructed by Atelocollagen-associated autologous chondrocyte implantation combined with anterior cruciate ligament reconstruction

**DOI:** 10.1186/s12891-020-03531-8

**Published:** 2020-07-27

**Authors:** Takuma Kaibara, Eiji Kondo, Masatake Matsuoka, Koji Iwasaki, Tomohiro Onodera, Daisuke Momma, Naoki Seito, Susumu Mikami, Norimasa Iwasaki

**Affiliations:** 1 Department of Orthopaedic Surgery, Hokkaido University Graduate School of Medicine, Kita-15, Nish-7, Kita-ku, Sapporo, Hokkaido 060-8638 Japan; 2 Center for Sports Medicine, Hokkaido University Hospital, Kita-15, Nish-7, Kita-ku, Sapporo, Hokkaido 060-8638 Japan; 3 Department of functional reconstruction for the knee joint, Hokkaido University, Kita-15, Nish-7, Kita-ku, Sapporo, Hokkaido 060-8638 Japan; 4 Department of Orthopaedic Surgery, Hokkaido Orthopaedic Memorial Hospital, 5-22, 7-Jo 13-Chome, Hiragishi, Toyohira-ku, Sapporo, Hokkaido 062-0937 Japan

**Keywords:** Osteochondral defect, Autologous chondrocyte implantation, Atelocollagen, Anterior cruciate ligament reconstruction, Orthopedics, Knee

## Abstract

**Background:**

Articular surface damage commonly associated with rupture of the anterior cruciate ligament (ACL). Large osteochondral defect, which consists of a severe depression fracture and a large cartilage defect, need to be treated due to deformation of the articular surface as it can impact the clinical outcome of ACL reconstruction. Although autologous chondrocyte implantation is one of the useful options in such cases, it can be questioned whether the reconstruction of the ACL and osteochondral defect should be performed in one procedure alone.

**Case presentation:**

We report a case of a 38-year-old male with a deep depression fracture extending to the edge of the lateral femoral condyle associated with ACL injury after twisting his right knee while skiing. The patient was successfully treated with tissue-engineered cartilage transplantation covered by the periosteum with an iliac bone graft combined with anatomic double-bundle ACL reconstruction. Histopathological examination of the transplanted cartilage taken at second-look arthroscopy showed a cartilage-like tissue in the middle to deep zone in which the extracellular matrix was largely stained with Safranin O. The patient was able to return to his previous level of skiing activity without any experience of knee pain. Magnetic resonance imaging at 4 years after surgery showed that the graft integrated to the border zone and subchondral bone. The operated knee showed negative Lachman test and had a full range of motion.

**Conclusions:**

To our knowledge, this is the first report of anatomic double-bundle ACL reconstruction with tissue-engineered cartilage transplantation and an iliac bone graft to restore the lateral edge of the femoral condyle.

## Background

The lateral femoral notch sign was first described by Loose et al. as a small depression fracture in the lateral femoral condyle associated with an anterior cruciate ligament (ACL) injury [1]. It is caused by an impression fracture of the lateral femoral condyle at the time of injury in 25% of patients [2]. Although the lateral femoral notch sign usually has only radiographic finding without the need for any surgical intervention, the severe depression fracture has clinical importance due to articular surface deformity of the femoral condyle as it can affect the clinical outcome of ACL reconstruction. However, there are several issues in such cases, we must be concerned with such as surgical strategies, options and the timing of ACL reconstruction [3–6].

A variety of surgical procedures have been developed to repair the articular surface of the knee. Treatment options for osteochondral defects of the knee include abrasion chondroplasty [7], debridement [8], drilling or microfracture [9], allograft transplantation [10], periosteal transplantation [11], and autograft transplantation [12]. Autologous chondrocyte implantation (ACI) was introduced in 1994 for the treatment of articular cartilage injuries [13] and favorable outcomes were continuously reported [14–16]. Recently, matrix-induced autologous chondrocyte implantation (MACI) that utilized the collagen carrier membrane has been attracting attention, especially for the large osteochondral lesion. Atelocollagen-associated ACI, one MACI technique, is a newly developed tissue-engineering approach for constructing a tissue-engineered cartilage in a three-dimensional culture using atelocollagen gel [17]. This technique is widely proposed as one option for the restoring osteochondral defect of the knee [18, 19]. However, it can be questioned whether the ACL reconstruction and the MACI procedure should be performed in one overall procedure.

Several issues need to be considered when making treatment decision for a full-thickness cartilage defect with an ACL injured knee. Only a few studies have described a combined treatment of osteochondral defects with ACL injured knee using autologous osteochondral grafting, autologous periosteum, periosteum-covered ACI, or MACI [20–23]. Although a combined treatment using ACI with ACL reconstruction seems to be one of the most useful options, there has been no previous report about a case of ACL injured knee with a huge depression fracture extending to the lateral wall of the femoral condyle.

Recently, we encountered a case of ACL injury with a huge depression fracture extending to lateral wall in the lateral femoral condyle. We treated the patient by using a transplantation method of tissue-engineered cartilage which used scaffold composed of atelocollagen gel matrix covered by the periosteum with an iliac bone graft combined with anatomic double-bundle ACL reconstruction. To our knowledge, this is the first report of ACL reconstruction with MACI implantation and iliac bone graft to reconstruct the lateral edge of the femoral condyle.

## Case presentation

**Fig. 1:**
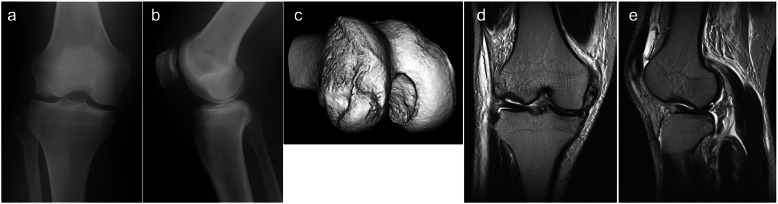
X-ray, computed tomography scan and magnetic resonance imaging before surgery. **a** Anteroposterior, **b** Lateral radiographs, **c** A 3-dimensional computed tomography of the right knee demonstrating a large depression fracture of the lateral femoral condyle. **d** Coronal T2-weighted, e Sagittal T2-weighted magnetic resonance imaging of the right knee taken before surgery demonstrated a large osteochondral defect of the lateral femoral condyle and superficial medial collateral ligament rupture

A 38-year-old male sustained an injury to his right knee while skiing. The patient experienced a twisting of his knee, while a valgus impaction force was applied to the slightly flexed knee. The patient had a dislocated feeling in his knee with severe knee pain and could not stand up. He visited our affiliate hospital on the day of the accident. Physical examination revealed knee effusion and a positive Lachman test with valgus instability in right knee. The patient presented the tenderness at the medial collateral ligament (MCL) and the lateral femoral condyle. The range of motion was reduced to 90 degrees of flexion due to knee pain. Fat droplets were present in the aspirated blood from the knee joint. Plain radiographs showed a huge depression fracture in the lateral femoral condyle (Fig. 1a, b). Computed tomography (CT) also revealed a huge depressed lesion of 35 mm × 15 mm in area and 7 mm in depth in the joint surface of the lateral femoral condyle extending to the edge of the condyle (Fig. 1c). Magnetic resonance imaging (MRI) showed an ACL and superficial MCL tears combined with a huge depression fracture in the injured knee (Fig. 1d, e).

Four weeks after injury, arthroscopy was performed at the same hospital. The arthroscopy revealed that the patient had an ACL rupture, a large cartilage injury, and a huge depression fracture on the lateral femoral condyle. Superficial MCL was repaired with a suture anchor. Eight weeks after injury, this patient was referred to our hospital. We decided a two-staged surgery which was ACL reconstruction with MACI implantation and an iliac bone graft. First, we conducted an arthroscopically assisted cartilage harvest for MACI procedure from the medial edge of the femoral trochlea of the ipsilateral knee joint [17]. Briefly, under general anesthesia, approximately 800 mg of cartilage tissue was harvested. The cartilage tissue was sent to the facility (Japan Tissue Engineering Co., Ltd., Gamagori, Japan). Chondrocytes were isolated from the obtained cartilage specimen and one volume of isolated chondrocytes was suspended into four volumes of atelocollagen solution (3% type I collagen; Koken, Tokyo, Japan). The mixture was put on culture flasks and gelated entirely by incubation at 37 °C. The tissue-engineered cartilage was then cultured in a three-dimension manner in an atmosphere of 5% carbon dioxide and 95% air at 37 °C for four weeks. The cultured cartilage had become a jelly-like form with the progress of cultivation [17–19].

Four weeks after harvest of the cartilage tissue, we carried out a second surgery. Under general anesthesia, the patient was placed in a supine position on the operating room table. First, an arthroscopic anatomic double-bundle ACL reconstruction with a hamstring tendon autograft was performed using the transtibial tunnel technique according to previous study [24]. The harvested ipsilateral semitendinosus tendon was halved and doubled over for graft preparation. A Leeds-Keio Artificial Ligament (Neoligament, Leeds, England) was mechanically connected at an unlooped end of the folded tendon [25, 26]. Then, an Endobutton CL BTB (Smith & Nephew, Andover, MA) was tied at the looped end [27].

**Fig. 2:**
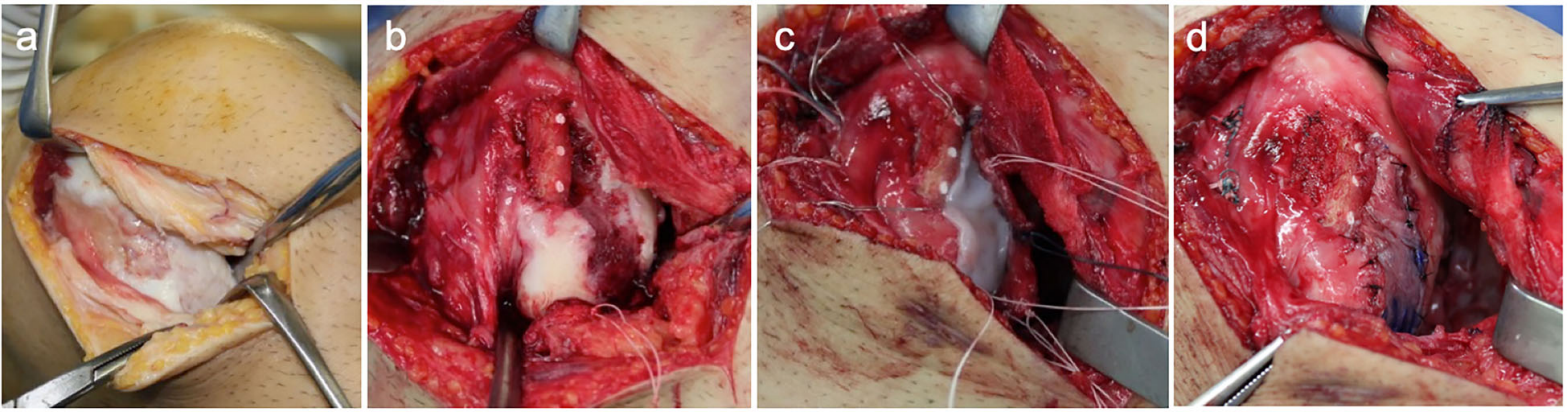
Surgical procedure for the large depressed fracture of the lateral femoral condyle. **a** The picture showing an extensive depressed osteochondral fracture on the lateral femoral condyle (approximately 20 × 30 mm). **b** After debridement and curettage of the lesion, an iliac cortical bone graft was fixed with three Poly-L-Lactic Acid pins. **c** Chondrocytes-atelocollagen gel was put on the defect area. d Iliac periosteum was sutured with 5–0 nylons to the surrounding rim

Second, the osteochondral defect and depression fracture of the lateral femoral condyle were exposed through the lateral parapatellar approach. The size of osteochondral defect was approximately 20 × 30 mm (Fig. 2a). The osteochondral lesion was debrided to expose the underlying subchondral plate. Bi-cortical bone block harvested from the right iliac crest was trimmed to reconstruct the normal shape of the lateral femoral condyle. The harvested bone block was fixed to the lateral edge of the lateral femoral condyle with three Poly-L-Lactic Acid pins (PL-Fix, Zimmer Biomet, Warsaw, IN) (Fig. 2b). The iliac periosteal flap was loosely sutured to the adjacent rim of the healthy cartilage with a polyester thread using a pull-out technique (Fig. 2c). After half the border of the periosteal flap was sutured, tissue-engineered cartilage covered by atelocollagen gel matrix was placed in the cartilage defect region, then the remaining half of the flap was sutured with a polyester thread and 5–0 nylon (Fig. 2d).

Finally, the graft for the PL bundle was placed through the PL tibial tunnel to the femoral tunnel with a passing pin. The graft for the AM bundle was introduced in the same way and fixed with 30 N tension to each graft using tensiometers (Yufu Itonaga Co., Ltd., Tokyo, Japan) at 10° of knee flexion. The 2 tape portions onto the tibia were secured using two spiked staples (Smith & Nephew) in the turn-buckle fashion. The operation time was 231 min.

**Fig. 3:**
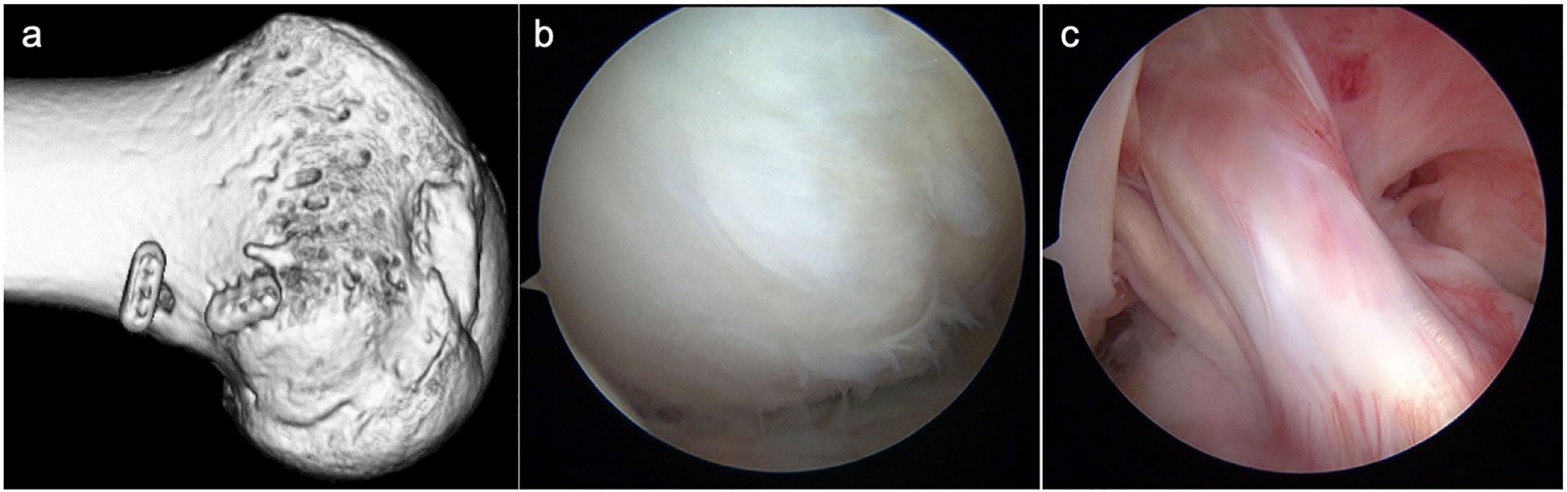
Postoperative imaging of computed tomography and second-look arthroscopic appearance. A 3-dimensional computed tomography of the lateral femoral condyle from a lateral side view at 13 months after surgery. **a** Postoperative image showed the contour of the lateral femoral condyle was reconstructed with the implanted iliac bone graft. **b** Smooth cartilaginous tissue with a slightly fibrillated surface was observed at the implantation site of the lateral femoral condyle. **c** Reconstructed anterior cruciate ligament grafts had no laceration or elongation and was wholly covered with synovium

**Fig. 4:**
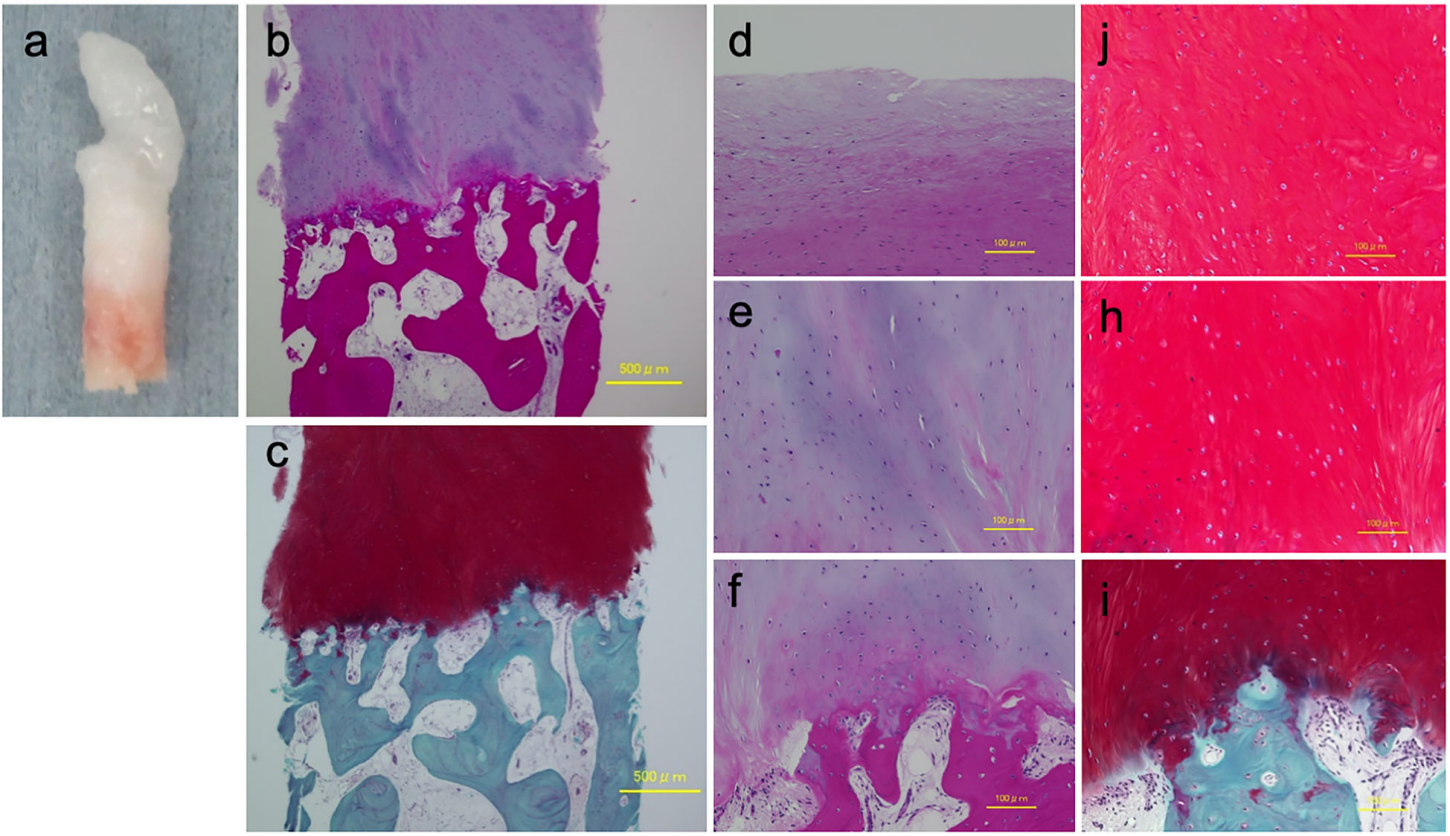
Histopathological findings of graft tissue taken by biopsy at 13 months postoperatively. **a** Gross view of the graft tissue showing cartilagenous tissue. Hematoxylin and eosin (**b**, **d-f**) and Safranin-O stain (**c**, **j-i**) at 4 × magnification (**b**, **c**) and 10 × magnification (**d**-**i**). Grafted tissue consisted of fibrous tissue in the superficial layer (**d**) and cartilage-like tissue in the middle to deep layer (**e**, **f**) in which the extracellular matrix was stained with Safranin O (**c**, **j**-**i**). Histologically, grafted tissue was well bonded to the subchondral bone (**f**, **i**), but the normal layer composition was not observed

After 2 weeks of the knee joint immobilization, continuous passive motion exercise of the knee joint was allowed. Partial weight-bearing was started at 6 weeks, and full weight-bearing was allowed from 8 weeks. A functional knee brace was used for a period of 3 months. CT scan taken 12 months after the 2nd surgery showed that the contour of the lateral femoral condyle was reconstructed with the implanted iliac bone graft (Fig. 3a). Thirteen months later, second-look arthroscopy and staple removal were performed. Second-look arthroscopy showed that the depressed area was covered with smooth cartilaginous tissue with a native cartilage-like hardness (Fig. 3b). ACL graft had no laceration and an excellent coverage of synovium (Fig. 3c). A needle biopsy was taken from the center of the grafted cartilage. The grafted cartilage histologically composed of fibrous tissue in the smooth superficial layer and cartilage-like tissue in the middle to deep layer where the extracellular matrix was strongly stained with Safranin O (Fig. 4). Implanted tissue had no vascularization and integrated into subchondral bone. The morphology of cells in the middle to deep layer were oval with a pericellular lacuna.

**Fig. 5:**
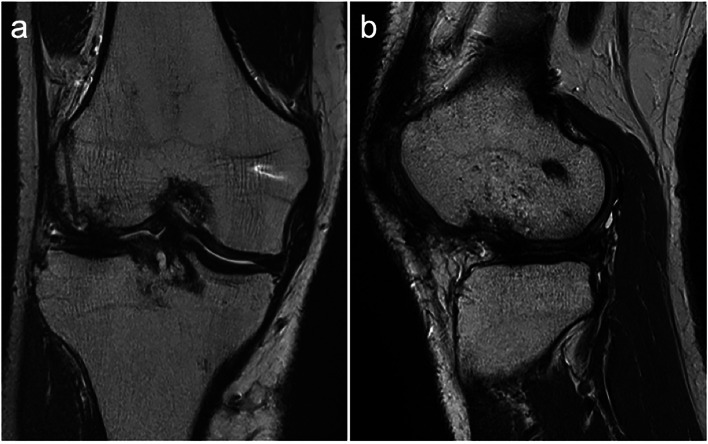
Magnetic resonance imaging (MRI) evaluation at 4 years after surgery. **a** Coronal T2-weighted, **b** Sagittal T2-weighted MRI images of the right knee demonstrating integration of graft tissue to border zone and subchondral bone

Sports activities were allowed at 18 months after surgery, and he was able to ski once again. The patient returned to his previous skiing activity level without any knee pain. After 4 years, MRI showed that the graft was incorporated into the border zone and the subchondral bone (Fig. 5). After a final follow-up, the knee showed a negative Lachman test, a negative pivot-shift test, and had a full range of knee motion. The side-to-side difference in the anterior laxity at 30 degrees of knee flexion measured with KT-2000 was 2.0 mm. Lysholm knee score was 94 points. The objective International Knee Documentation Committee (IKDC) was determined as grade A. Knee injury and Osteoarthritis Outcome Score (KOOS) was 82.1, 94.4, 100.0, 50.0, and 68.8 in Pain, other Symptoms, Function in daily living (ADL), Function in Sport and Recreation (Sport/Rec), and knee-related Quality of Life (QOL), respectively.

## Discussion

We reported a case with a large cartilage defect and deep depression fracture in the femoral condyle extending to the lateral edge associated with acute ACL injury. Because the lateral femoral condyle could not be reconstructed by transplanting a tissue-engineered cartilage, the bi-cortical bone block and the iliac periosteum from the iliac crest was applied to achieve the anatomical contour of the lateral femoral condyle.

There were several key issues in this case. As for ACL reconstruction, we believe that the anatomic double-bundle technique was the best way to restore normal knee function [24, 28], although the clinical utility of anatomic double-bundle reconstruction in comparison with the conventional single-bundle reconstruction remains controversial [29]. In the current case, we performed anatomic double-bundle procedure, lateral wall reconstruction, and MACI implantation in the lateral femoral condyle simultaneously to avoid the ACI graft failure due to the knee joint instability. As for depression fracture in the lateral femoral condyle, the transplantation of iliac cortical bone was needed to reconstruct a normal contour of the lateral wall because the lateral femoral condyle was severely depressed at the time of injury. This type of operation offers several advantages such as enhanced articular cartilage repair, early discharge and a daily and sports-related activities although it might require a longer operation time resulting in an increased invasiveness to the knee. Therefore, the surgeon needs not only technical skill but also must proceed in a step by step process.

Various type of ACI have been investigated for the treatment for osteochondral defects of the knee [30, 31]. Atelocollagen-associated ACI utilized atelocollagen as a matrix for the three-dimensional expansion and embedding of harvested cartilage [17]. Previous study reported that human chondrocytes can proliferate and synthesize the extracellular matrix without changing their phenotype in atelocollagen gel for 4 weeks [32]. Several clinical trials have confirmed the efficacy of atelocollagen-associated ACI [18, 19, 33]. This atelocollagen-associated ACI procedure is the only ACI procedure which has been approved by the Japanese Health Insurance since 2013. In current case, the histology revealed that the repaired tissue consisted of a hyaline-like cartilage with a rich extracellular matrix although the zonal arrangement of the repaired tissue were not completely well-organized.

Amin et al. compared the clinical results of ACI in combination with ACL reconstruction to ACI following previous ACL reconstruction in their patients and concluded that the treatment of ACI in combination with ACL reconstruction was technically achievable, with good to excellent outcomes over a short time period [20]. Dhinsa et al. reported that ACI in combination with ACL reconstruction is a feasible option with similar results as those patients who have had the procedures staged and prioritized a combined strategy in terms of cost and a short rehabilitation period [21]. Pike et al. reported improved clinical outcomes of eighteen out of twenty-six patients with concurrent ACI and either primary or revision ACL reconstruction with a mean follow-up of 7 years and concluded that ACI can contribute to moderately improved pain and function during a long-term follow-up [23]. Adachi et al. reported an osteochondral lesion (15 × 15 mm) on the lateral femoral condyle reconstructed by the transplantation of tissue-engineered cartilage combined with an iliac bone graft [34]. We referred to this technique. Based on these studies, in case of an ACL injured knee with a large cartilage defect and a huge depression fracture extending to the lateral wall, a surgical plan should be discussed for each case in advance whether it be a one-staged or a two-staged procedure.

There are several limitations in this report. First, a limitation of this ACI procedure is that a restricted number of autologous chondrocytes can be obtained from any one patient. A second disadvantage is that a 2-stage operation is required. Moreover, arthrotomy has to be performed for second implantation, and this is an invasive procedure, although the ideal chondrocyte implantation procedure is still controversial. The third limitation is the regenerated tissue after our procedure. Because the histological evaluations demonstrated that the regenerated tissues were not true hyaline cartilage and that columnar distribution of the normal cartilage was not established, there is a possibility that longer follow-up would show that the regenerated tissue deteriorates.

In conclusion, although future studies are needed to investigate the reproducibility of our surgical strategy, simultaneous cartilage repair with atelocollagen-associated ACI and lateral wall reconstruction with an iliac bone graft for femoral large bony defect combined with ACL reconstruction had favorable four-year outcomes.

## Data Availability

This is a case report of a single patient, in order to protect privacy and respect confidentiality; none of the raw data has been made available in any public repository. The original operation reports, intraoperative photographs, imaging studies and outpatient clinical records are retained as per normal procedure among the medical records of our institution. All data concerning the case are presented in the manuscript.

## Abbreviations

ACL: Anterior cruciate ligament
ACI: Autologous chondrocyte implantation
CT: Computed tomography
MCL: Medial collateral ligament
MRI: Magnetic resonance imaging
AM: Anterior-medial
PL: Postero-lateral
